# Bacillary angiomatosis with bone invasion*

**DOI:** 10.1590/abd1806-4841.20165436

**Published:** 2016

**Authors:** Lucia Martins Diniz, Karina Bittencourt Medeiros, Luana Gomes Landeiro, Elton Almeida Lucas

**Affiliations:** 1 Universidade Federal do Espírito Santo (UFES) – Vitória (ES), Brazil

**Keywords:** Acquired immunodeficiency syndrome, Angiomatosis, bacillary, Bartonella, Bartonella quintana

## Abstract

Bacillary angiomatosis is an infection determined by *Bartonella henselae* and *B. quintana*, rare and prevalent in patients with acquired immunodeficiency syndrome. We describe a case of a patient with AIDS and TCD4+ cells equal to 9/mm^3^, showing reddish-violet papular and nodular lesions, disseminated over the skin, most on the back of the right hand and third finger, with osteolysis of the distal phalanx observed by radiography. The findings of vascular proliferation with presence of bacilli, on the histopathological examination of the skin and bone lesions, led to the diagnosis of bacillary angiomatosis. Corroborating the literature, in the present case the infection affected a young man (29 years old) with advanced immunosuppression and clinical and histological lesions compatible with the diagnosis.

## INTRODUCTION

Bacillary angiomatosis is an infection universally distributed, rare, caused by Gram-negative and facultative intracellular bacilli of the *Bartonella* genus, which 18 species and subspecies are currently known, and which also determine other diseases in man.^1^ The species responsible for bacillary angiomatosis are *B. henselae* and *B. quintana*. Cats are the main hosts of *B. henselae*, transmitting the bacillus to man through bites and scratches or flea bites. *B. quintana* has homeless men as hosts and its transmission is through bites of lice present on human skin.^2^

Patients most affected by the disease are the carriers of acquired immunodeficiency syndrome with CD4+ cell counts below 200/mm³, but bacillary angiomatosis can also be evidenced in immunosuppressed by other causes, such as lymphomas, leukemias and immunotherapeutic drugs, and rarely in immunocompetent subjects.^1,3^

The infection presents systemic dissemination, affecting more often the skin, but also the bones, lymph nodes and viscera (liver, spleen, brain and gastrointestinal and respiratory tracts).^4^ In skin, it can be observed isolated papules or red erythematous or purpuric nodules, single or multiple, with soft or firm consistency, accompanied by fever, anorexia, weight loss, abdominal pain, nausea, vomiting, diarrhea, etc.^1^

Histology of the lesions of affected organs shows vascular proliferation, hence the name "angiomatosis". By silver staining, the presence of the bacilli is revealed, thus "bacillary".^4^ In histological description, capillary proliferation is observed characteristically in lobes – central capillaries are more differentiated and peripheral capillaries are less mature – with lumens not so evident. There are also several mitoses and cell atypias, in addition to leukocytes and leukocytoclasia in the interstices of lobes of lesions without ulceration. Capillaries are arranged around the bacillary clusters, evident in staining with hematoxylin-eosin and silver.^1^

The main differential diagnosis is with Kaposi's sarcoma. One should also consider pyogenic granuloma, lymphomas, atypical mycobacterioses na agiomas.^1^

In the treatment of infection is used erythromycin (500 mg, four times daily) or doxycycline (100 mg twice daily) for eight to 16 weeks.^5^

## CASE REPORT

**Figure 1:**
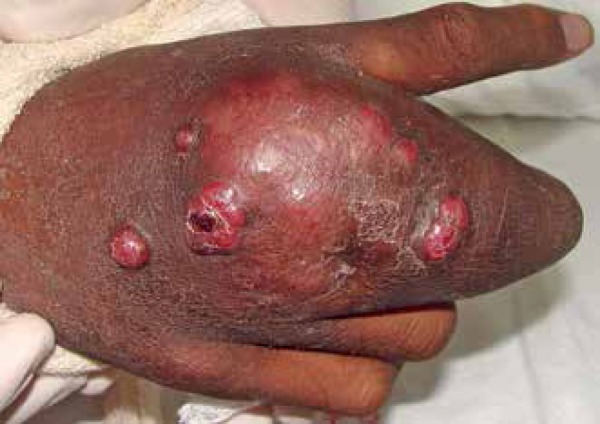
Violaceous and erythematous nodules over tumor on the back of the right hand and third finger

**Figure 2:**
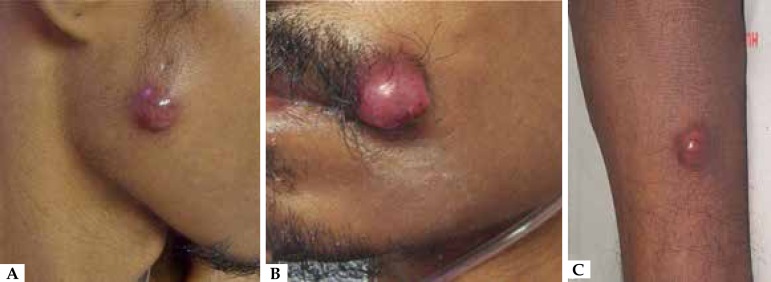
Violaceous and erythematous nodule on the right parotidomasseteric region (**A**), left labial commissure (**B**) and A B C right leg (**C**)

**Figure 3:**
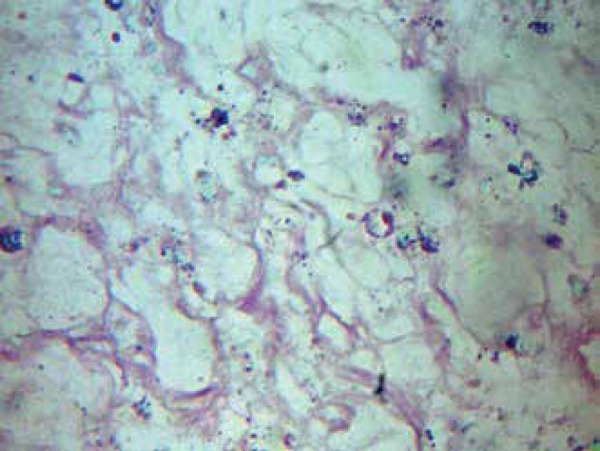
Histopathological examination of the skin (epigastric lesion) showing bacilli aggregate (silver, 100x)

**Figure 4:**
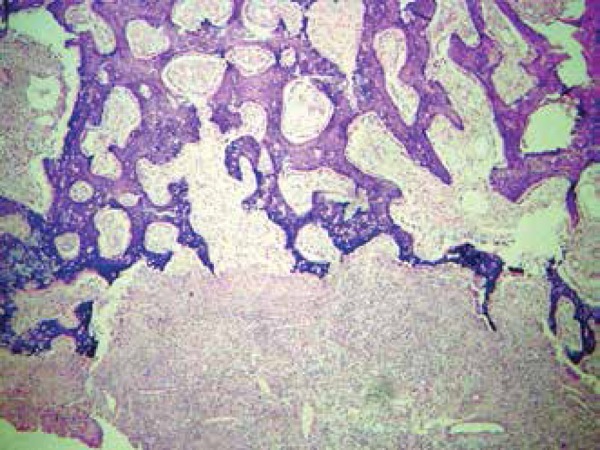
Histopathological examination of a bone fragment of the finger with vascular proliferation partially involving the trabecular bone and the presence of bacilli (hematoxylin and eosin, 40x)

**Figure 5:**
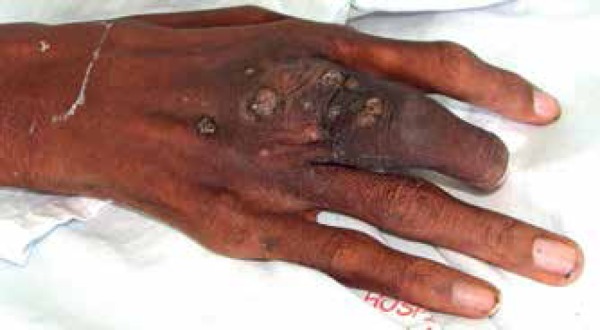
Right hand aspect after 30 days of treatment with azithromycin and ceftriaxone. There is a reduction in bone volume and regression of overlying cutaneous lesions

**Figure 6:**
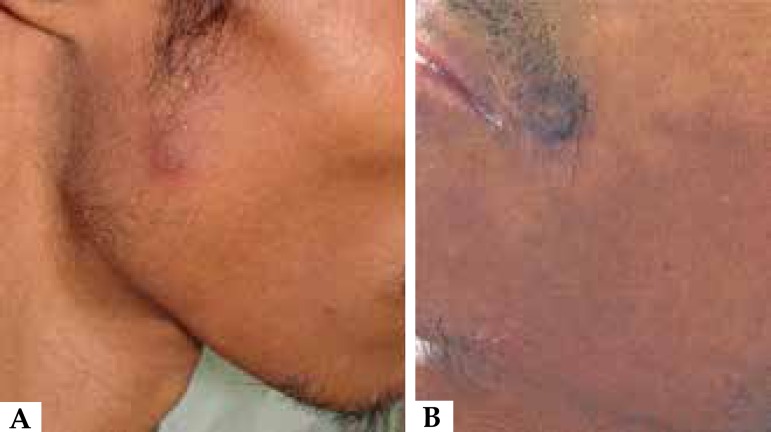
Aspect of skin lesions on the right parotidomasseteric region (**A**) and left labial commissure (**B**) after 30 days of treatment with azithromycin and ceftriaxone

Man, 29 years, black, presented for a year tumor with overlapping violaceous erythematous nodules located on the back of the hand and the third right finger (Figure 1). During evolution, violaceous erythematous nodules appeared on the right parotidomasseteric region, left labial commissure, chest, abdomen, legs and feet (Figure 2). He began to present weight loss, prostration, fever and apathy and was admitted to the emergency room of a university hospital. The research of the human immunodeficiency virus was positive. The viral load was 11,398 copies, and the CD4 + T cells count was 9/mm^3^. A skin assessment was requested to the Dermatology service, which proceeded to the biopsy of the abdomen lesion, suspecting of Kaposi's sarcoma or bacillary angiomatosis. Histopathological examination showed multiple vascular proliferations with interposed neutrophils. Silver staining showed bacilli aggregates, leading to the diagnosis of bacillary angiomatosis (Figure 3). Radiography of the right hand showed lytic lesion in the distal phalanx of the third finger. Histopathology of this lesion showed vascular proliferation partially involving the trabecular bone with bacilli inside, stained with hematoxylin and eosin, characterizing it as bacillary angiomatosis (Figure 4). The other tests, HBsAg, anti-HCV and syphilis were negative, and PPD was non-reactive. CT scans of the chest and abdomen did not indicate visceral involvement. The patient received azithromycin and ceftriaxone, and complete regression of cutaneous lesions was observed after 30 days (Figures 5 and 6). When the patient was clinically stable antiretroviral therapy was started and the treatment was supplemented to bacillary angiomatosis with doxycycline (200 mg daily) for three months.

## DISCUSSION

Bacillary angiomatosis is a rare infection in patients with AIDS.^1^ Gazineo *et al.*, studying the cases of bacillary angiomatosis in patients with AIDS in five referral centers of Rio de Janeiro between 1992 and 1997 found 1.42 cases per 1,000 patients.^4^ In Germany, Plettenberg *et al.* reported 1.2 cases per 1,000 HIV-positive patients between 1990 and 1996.^6^

Bacillary angiomatosis is observed late in patients with AIDS. Opportunistic disease appears in individuals with advanced immunosuppression, usually with CD4 + cell counts below 200/mm^3^.^1^ Gazineo *et al.*^4^ observed the median of TCD4 + cells equal to 85.8 (± 73.9) cells/mm^3^. Plettenberg *et al.*^6^ and Mohle-Boetani *et al.*^7^ found median of 30 cells/mm^3^ and 21 cells/mm^3^, respectively. The patient in this report had 9 cells/mm^3^, namely severe immunosuppression.

Patients with this infection described in the medical literature are men aged between 35 and 39 years - profile compatible with patients most affected by AIDS.^4,6,7^ The patient described here was a man, but younger (29 years).

The skin is the organ most affected by bacillary angiomatosis, presenting violaceous erythematous lesions, papular, nodular or tumor, single or multiple,^1^ as presented by the patient described. Bone involvement is characterized by well circumscribed osteolytic lesions, painful, cortical or periosteal, which mainly affect the long bones and are observed in X-rays. *B. quintana* is associated more often with bone changes.^8^

The bacillary angiomatosis has as differential diagnosis the Kaposi's sarcoma. In this differentiation is necessary histological evidence, which may be defined by an experienced pathologist with the use of staining with hematoxylin-eosin for the finding of bacilli, epithelioid cells and well-formed blood vessels without fusiform fascicles.^9,10^ Research centers use culture of skin material, serology, indirect immunofluorescence and polymerase chain reaction.^1^

It was not possible to identify the species of *Bartonella* in this case. As the patient denied contact with cats, living in precarious conditions, presenting osteolytic lesions and deep nodules, possibly he was infected by *B. Quintana*.

Prutzky *et al.*^5^ performed a systematic review and meta-analysis of the treatment of bacillary angiomatosis and showed no statistical difference in cure and relapse rates in comparison between erythromycin and doxycycline, but there was statistical difference in the cure rate, although not in the relapse rate in comparison of erythromycin with amoxicillin-clavulanate, cefuroxime and imipenem. The importance of early recognition of this disease lies in the fact that it is potentially fatal, but easily treatable.^1^

To date, Brazilian medical literature recorded 17 reported cases of bacillary angiomatosis,^2^ one in HIV-negative patient and three in patients with skin and bone lesions.^4^ The case reported adds to cases of skin and bone involvement and is the first described in the state of Espírito Santo.
